# Neonates presenting with severe complications of frenotomy: a case series

**DOI:** 10.1186/1752-1947-6-77

**Published:** 2012-03-06

**Authors:** Peace I Opara, Nneka Gabriel-Job, Kingsley O Opara

**Affiliations:** 1 Department of Pediatrics, University of Port Harcourt Teaching Hospital, Port Harcourt, Nigeria; 2 Plastic Surgery Unit, Department of Surgery, Imo State University Teaching Hospital, Orlu, Nigeria

## Abstract

**Introduction:**

Tongue-tie or ankyloglossia is an anatomic variation in which the lingual frenulum is thick, short or tight. It may be asymptomatic, or present with complications like breast feeding difficulties or speech, dental and cosmetic problems. The treatment of this condition, where indicated, is frenotomy. This procedure usually has few or no complications. However, when it is done by untrained personnel, it may lead to life-threatening complications. This paper highlights complications that could arise from improper treatment of ankyloglossia.

**Case presentation:**

Case 1 was a one-day-old male neonate, a Nigerian of Igbo ethnicity, who was admitted with bleeding from the mouth and passage of dark stools after clipping of the frenulum by a traditional birth attendant. He was severely pale and in hypovolemic shock, with a severed frenulum which was bleeding actively. His packed cell volume was 15%. He was resuscitated with intravenous fluids and a blood transfusion. The bleeding was controlled using an adrenaline pack. He also received antibiotics. He was discharged five days later.

Case 2 was a three-day-old male neonate, a Nigerian of Ikwerre ethnicity, who was admitted with profuse bleeding from a soft tissue injury under the tongue, after clipping of the frenulum by a community health worker. He was severely pale and lethargic. He was resuscitated with intravenous fluids and a blood transfusion. The bleeding vessel was ligated with repair of the soft tissue. He also received antibiotics and was discharged home one week later.

**Conclusion:**

Treatment of tongue-tie, a benign condition, when done by untrained personnel may result in life-threatening complications. Clinicians should pay more attention to parents' worries about this condition and give adequate counseling or refer them to trained personnel for surgical intervention where clinically indicated.

## Introduction

Tongue-tie (ankyloglossia) is a congenital condition, often hereditary, in which the lingual frenulum is abnormally short and may therefore restrict mobility of the tongue tip [1,2]. In this condition, the lingual frenulum, a normal structure present in all babies, tethers the tongue to the floor of the mouth thereby preventing extension of the tongue beyond the lower gum.

Reported prevalence varies from 1.7% to 10.7% with a male predominance [2-5]. Persistence of the condition is uncommon beyond two to three years of age when compared with the higher incidence in neonates, suggesting a lessening of the degree of the abnormality with growth and development [6].

Tongue-ties can be symptomatic or asymptomatic. Signs of symptomatic tongue-tie include a notched or heart-shaped tongue tip, a flattened or square-shaped tongue tip when the tongue is extended, inability to move the tongue sideways and inability of the tongue to touch the roof of the mouth or extend beyond the lips [7]. Certain complications are associated with tongue-tie, the earliest of which are breastfeeding difficulties. An infant's inability to extend the tongue beyond the lower gum margins results in breastfeeding problems, including sore nipples and poor milk supply for the mother and poor weight gain for the baby [8]. Other problems associated with significant tongue-tie include speech articulation disorders, poor oral hygiene, dental problems and various social problems [9].

There is continuing controversy over the treatment of tongue-tie dating back many centuries. Before the 19th century, midwives were reported to have kept sharp finger nails to slash the membrane under the tongue of all newborns [10]. Complications of this procedure included bleeding, infection and injury to the muscles under the tongue. Where treatment is indicated, and properly done by trained clinicians, the procedure (frenotomy) has few or no complications and can be done without anesthesia [1,2,4].

The clipping of the frenulum without reason, which was common practice in earlier years, resulted in the surgical treatment of tongue-tie falling into disrepute amongst many in the medical community [1,2,10]. This unwillingness of many clinicians to intervene surgically has, however, led some parents in our environment to seek help from unqualified medical and non-medical personnel; thus contributing to the morbidity associated with treatment.

The aim of this paper is to highlight complications that may arise from improper treatment of tongue-tie by unqualified personnel.

## Case presentation

### Case 1

A one-day-old male Nigerian neonate of Igbo ethnicity was referred from a private hospital with complaints of bleeding from the mouth and vomiting of blood shortly after delivery. Frank bleeding started about an hour after delivery. There was no bleeding from any other site. He had vomited nine times on the first day of life and three times on the day of presentation.

He had received some antibiotic injections from the referring clinic but bleeding had persisted. A diagnosis of neonatal sepsis with disseminated intravascular coagulopathy was made and he was referred to our hospital.

The pregnancy had been supervised in a maternity home and had been uneventful. Delivery was at term by a traditional birth attendant (TBA). Further history revealed that the lingual frenulum had been clipped by the TBA about an hour after birth, after which bleeding had commenced. The indication for frenotomy could not be ascertained.

The baby was the last of five children, four girls and a boy. All the other children had undergone the same procedure, performed by the same TBA, within the first few hours of birth, with minimal complications. The family was of a low socioeconomic background.

On examination, our patient was in respiratory distress, severely pale and had cool extremities. He was noted to have a bleeding severed frenulum. His peripheral pulses were weak and thready and his systolic blood pressure was unrecordable. His heart rate was 180 beats per minute with normal heart sounds. His respiratory rate was 94 cycles per minute and breath sounds were vesicular. His abdomen was flat and soft with no palpable organs. He was conscious but lethargic, with a depressed anterior fontanelle. His primitive reflexes were depressed but he had normal tone in the limbs. A review of his systems showed that he was very weak, had poor suck and had not passed urine in the last eight hours.

A diagnosis was made of severe anemia and hypovolemic shock secondary to acute blood loss, occurring after the clipping of the frenulum. An adrenaline pack was applied to the bleeding site and bleeding was controlled within a few minutes. His urgent packed cell volume (PCV) was 15%.

He was rehydrated with normal saline until blood was available for transfusion. He received whole blood transfusion and a single volume exchange blood transfusion. He was commenced on broad spectrum antibiotics (ceftazidime). Vitamin K injections were also given and his vital signs were monitored. His post-transfusion PCV was 45%. His electrolytes, urea and creatinine results were normal except for elevated urea; his clotting profile was also within normal limits. He also developed jaundice which responded to phototherapy. He was discharged five days after admission.

### Case 2

A three-day-old full-term male Nigerian neonate of Ikwerre ethnicity was rushed into our children's emergency ward with a history of profuse bleeding from the mouth. Bleeding followed clipping of the frenulum done by a community health worker, a few hours prior to presentation.

The pregnancy, labor and delivery had been supervised in a primary health care centre and had been uneventful. The baby had started breastfeeding, but was thought to be breastfeeding poorly because of tongue-tie. He was therefore taken to the community health worker on the third day of life, who severed the lingual frenulum using a new razor blade. He started bleeding from the site and was rushed to our institution by the community health worker, a few hours after attempts at controlling the bleeding using pressure dressings had failed.

At presentation he was bleeding profusely from the mouth and severely pale. The frenulum had been severed with obvious soft tissue injury and a bleeding vessel under the tongue. He was in respiratory distress with respiratory rate of 80 cycles per minute, but was not cyanosed and had normal breath sounds. His heart rate was 172 beats per minute with normal heart sounds. He was conscious but lethargic, the anterior fontanel was depressed and his primitive reflexes were normal. There were no significant findings in his abdomen.

Pressure was applied to the bleeding point with an adrenaline pack, while the surgeons were called in. The bleeding vessel was ligated and the soft tissue injury repaired. His urgent PCV was 18% and he was transfused with whole blood and a single volume exchange blood transfusion. He was commenced on broad spectrum antibiotics and vitamin K injections. His post-transfusion PCV was 36%; blood cultures yielded no growth and he was discharged home one week after admission.

## Discussion

Anatomical restraining of tongue movement (tongue-tie, ankyloglossia) has been known for centuries. Heated debates, however, still persist on its clinical significance and indications for treatment. Most authorities in the field of infant feeding and lactation agree that breastfeeding problems, such as nipple pain and latching difficulties, are common signs of clinically significant tongue-tie and indications for frenotomy, while the sole presence of a visible lingual frenulum is not [11]. One of our patients had been taken to seek help because of the complaint of poor breastfeeding.

Studies show that ankyloglossia in infants is associated with a 25% to 60% incidence of difficulties with breastfeeding, such as failure to thrive, maternal nipple damage, maternal breast pain, poor milk supply, breast engorgement and refusing the breast [12-14]. In order for an infant to properly latch on to the mother's breast, the infant's tongue must be able to thrust to the edge of the lower gum and cup around the areola and the mother's elongated nipple [4]. The ineffective latch caused by ankyloglossia has been postulated as one of the primary underlying causes of all of the listed problems [12-14].

The patient in Case 1 had no given symptoms to indicate frenotomy, except as part of a routine clipping of the tongue which had been done by the same TBA for all his siblings. This practice, which was quite common in the mid-19th century where midwives kept a sharp fingernail to routinely clip the lingual frenulum of all newborns [10], may still be prevalent in some cultures and societies in our environment, as portrayed by this case.

There is no accepted, widely used method for diagnosing ankyloglossia [8]. Many authors use criteria based on the physical characteristics of the infants' oral anatomy [3,8,12,14]. Commonly employed criteria include the frenulum being abnormally short and thick, causing the tongue to become heart-shaped upon protrusion; signs of functional impairment, such as an inability to protrude the tongue past the gum line; and other indications of decreased tongue mobility [4]. None of these criteria have been validated [4]. Our patients were seen post-treatment, so whether or not they had significant tongue-tie could not be ascertained. A previous Nigerian study, however, showed that diagnostic accuracy by traditional and orthodox healthcare providers was very low, whilst parental curiosity and myth about tongue-tie were high [15].

The most common treatment of infant ankyloglossia is simple frenotomy [4,14]. Frenotomy, depending on the type of ankyloglossia, may be accomplished by incising 2 to 3 mm into the lingual frenulum, care being taken not to incise any vascular tissue (for example, the base of the tongue, the genioglossus muscle or the gingival mucosa). There should be minimal blood loss, that is, no more than a drop or two, collected on sterile gauze [14]. However, when this procedure is done by untrained personnel as was the case in our patients, severe complications may arise. These include bleeding and soft tissue injury as seen in our patients, and others such as submandibular orifice injury. Similar complications, including infections, were reported in Benin City, Nigeria [15], when attempts at tongue release were carried out by untrained personnel. It is also important to note that a severed frenulum as a cause of bleeding in a newborn requires a high index of suspicion to reduce associated morbidity. In one of our patients, this was missed at the first point of care before he was referred to our hospital.

## Conclusion

Ankyloglossia is a relatively benign condition with a simple method of correction (frenotomy). In untrained hands, attempts at release could result in life-threatening complications. There is the need for public enlightenment on the condition. Clinicians should also pay more attention to parental worries about tongue-tie and intervene where indicated. There is also a need for a high index of suspicion of a severed frenulum when neonates present with bleeding from the mouth.

## Consent

Written informed consents were obtained from the parents of the neonates for publication of this case series. A copy of the written consent is available for review by the Editor-in-Chief of this journal.

## Competing interests

The authors declare that they have no competing interests.

## Authors' contributions

PIO and NG were part of the managing team and were involved in the concept and design of the reports. All authors were involved in, and contributed equally to drafting the manuscript and participated in reading and revising the manuscript. All authors read and approved the final manuscript.
